# Stability of a Dumbbell Micro-Swimmer

**DOI:** 10.3390/mi10010033

**Published:** 2019-01-07

**Authors:** Takuji Ishikawa

**Affiliations:** 1 Department Finemechanics, Graduate School of Engineering, Tohoku University, Miyagi Prefecture 980-8579, Japan; ishikawa@bfsl.mech.tohoku.ac.jp; Tel.: +81-22-795-4009; 2 Graduate School of Biomedical Engineering, Tohoku University, Miyagi Prefecture 980-8579, Japan

**Keywords:** squirmer, locomotion, Stokes flow, hydrodynamic interaction, linear stability analysis, boundary element method

## Abstract

A squirmer model achieves propulsion by generating surface squirming velocities. This model has been used to analyze the movement of micro-swimmers, such as microorganisms and Janus particles. Although squirmer motion has been widely investigated, motions of two connected squirmers, i.e., a dumbbell squirmer, remain to be clarified. The stable assembly of multiple micro-swimmers could be a key technology for future micromachine applications. Therefore, in this study, we investigated the swimming behavior and stability of a dumbbell squirmer. We first examined far-field stability through linear stability analysis, and found that stable forward swimming could not be achieved by a dumbbell squirmer in the far field without the addition of external torque. We then investigated the swimming speed of a dumbbell squirmer connected by a short rigid rod using a boundary element method. Finally, we investigated the swimming stability of a dumbbell squirmer connected by a spring. Our results demonstrated that stable side-by-side swimming can be achieved by pullers. When the aft squirmer was a strong pusher, fore and aft swimming were stable and swimming speed increased significantly. The findings of this study will be useful for the future design of assembled micro-swimmers.

## 1. Introduction

Artificial micro-swimmers have attracted strong research interest due to their potential environmental and medical applications [1,2]. The motility of micro-swimmers enables them to manipulate objects, carry chemicals, and react to their environment; therefore, they can be utilized in therapeutic treatments, sensing, and environmental remediation. To date, a wide variety of propulsion mechanisms have been proposed to generate motility in micro-swimmers, including magnetic forces, chemical forces, optical forces, bubbles, ultrasound, and fluid oscillation [3,4,5,6,7,8]. Recently, some studies have attempted to establish an assembly of micro-swimmers to generate stronger forces or large-scale mixing [9,10,11,12,13]. The stable assembly of multiple micro-swimmers could be a key technology for future micromachine applications. Hydrodynamic forces play an important role in constructing such assemblies, because the stability of an assembled micro-swimmer is strongly influenced by the flow field generated by surrounding micro-swimmers. However, the hydrodynamic effects at work in micro-swimmer assemblies remain to be clarified.

The flow field around a micro-swimmer can be described by Stokes flow, i.e., a negligible inertial effect, given that the Reynolds number based on the scale and speed of the micro-swimmer and the kinetic viscosity of the surrounding fluid often becomes much less than unity. Purcell [14] showed that simple reciprocal body deformation cannot induce micro-swimmer migration in a Stokes flow regime; this is known as the scallop theorem. To overcome the implications of the scallop theorem, the micro-swimmer must undergo a nonreciprocal body deformation or an external force must be introduced. In a Stokes flow regime, flow disturbance induced by a force decays inversely proportional to the distance, such that the hydrodynamic interaction propagates even in the far field. Mathematical frameworks to analyze the hydrodynamics of artificial and natural micro-swimmers, as well as their interactions, have been elaborated on in recent review papers [15,16,17,18,19,20].

One of the most popular mathematical micro-swimmer models may be the squirmer model, which was first proposed by Lighthill [21] and extended by Blake [22]. The squirmer model propels itself by generating surface squirming velocities. The model was originally proposed for ciliates and microalgae, such as *Opalina* and *Volvox*, and reproduces the flow field around *Volvox* well [23]. The squirmer model has also been used to analyze the motion of Janus particles [24] and self-propelling liquid droplets [25]; thus, it has been accepted as a general model for micro-swimmers. The squirmer model has been used to analyze nutrient uptake [26,27,28], swimming efficiency [29,30], two-body hydrodynamic interactions [31,32,33], collective swimming [34,35,36], self-diffusivity [37], and rheology [38]. For details of the squirmer model, please refer to the excellent review by Pedley [39].

Although these previous studies have clarified various aspects of the squirmer model, the motion of two assembled squirmers, i.e., a dumbbell squirmer, has not yet been investigated in detail. In a previous study [31], we calculated hydrodynamic interactions between two freely swimming squirmers. Götze and Gompper [32] also investigated two freely swimming squirmers by considering thermal fluctuation. Navarro and Pagonabarraga [33] investigated the hydrodynamic interactions of two trapped squirmers. However, none of these studies examined free-swimming dumbbell squirmers. Understanding the swimming behaviors and stability of the dumbbell squirmer is an important next step in constructing a large assembly of micro-swimmers. Therefore, in this study, we investigated the swimming behaviors and stability of the dumbbell squirmer.

In Section 2, we explain the squirmer model, basic equations, and numerical methods. In Section 3, we investigate the stability of the dumbbell squirmer in the far field using linear stability analysis. In Section 4, we discuss the swimming speed of a dumbbell squirmer connected by a short rigid rod, using a boundary element method. In Section 5, we discuss the swimming behaviors and stability of a dumbbell squirmer connected by a spring. Our conclusions are presented in Section 6.

## 2. Basic Equations and Numerical Methods

### 2.1. Squirmer Model

The squirmer model used in this study is a spherical steady squirmer. The squirmer has a radius *a*, and is assumed to be neutrally buoyant and non-Brownian. The surface of the sphere moves purely tangentially with respect to the cell body; this tangential motion is axisymmetric and time-independent. The tangential surface velocity **u***^s^* is given by

(1) $$u^{s}=\sum_{n=1}^{2}\frac{2}{n\left(n+1\right)}B_{n}\left(\frac{e\cdot r}{r}\frac{r}{r}-e\right)P_{n}^{\prime }\left(e\cdot r/r\right)$$

where *P_n_* is the *n*th Legendre polynomial, **e** is the unit orientation vector of the squirmer, and **r** is the position vector, *r* = |**r**|. A solitary squirmer swims at speed *U*_0_ = 2*B*_1_/3. As in our previous studies [28,34,35], we omitted squirming modes higher than the second (i.e., *B_n_* = 0 when n≥3) because the effect of higher modes becomes negligibly small compared to that of the first and second modes in far-field hydrodynamic interactions.

We denote the ratio of second mode to first mode squirming as *β*, so that we have *β* = *B*_2_/*B*_1_. A squirmer with positive *β* is a puller, analogous to a micro-swimmer for which thrust is mainly generated in front of the body. A squirmer with negative *β* is a pusher, analogous to a micro-swimmer for which thrust is mainly generated behind the body. A squirmer with *β* = 0 is a neutral swimmer, analogous to a micro-swimmer for which the thrust and drag centers coincide.

### 2.2. Basic Equations

Due to the small size of a micro-swimmer, we neglect the inertial effects on fluid flow. In a Stokes flow regime, the velocity around two squirmers can be expressed by a boundary integral equation, as follows [31]:

(2) $$u\left(x\right)=-\frac{1}{8\pi \mu }\sum_{m=1}^{2}\int J\left(x-y\right)\cdot q\left(y\right)\;dA_{m}\left(y\right)$$

where *μ* is the viscosity and *A_m_* is the surface of squirmer *m*. **J** is the Green’s function for unbounded fluid given by $J_{ij}=\delta_{ij}/r+r_{i}r_{j}/r^{3}$, where **r** = **x** – **y**, and **δ** is the Kronecker delta. The single-layer potential **q** is found by subtracting the traction force on the inner surface, **f***_in_*, from that on the outer surface, **f***_out_*, i.e., **q** = **f***_out_* − **f***_in_*. The boundary condition is given by:

(3) $$u\left(r\right)=U_{m}+\Omega_{m}\wedge \left(r-r_{m}^{c}\right)+u_{m}^{s}\;,\;r\in A_{m}$$

where **U***_m_* and **Ω***_m_* are the translational and rotational velocities of squirmer *m*, $r_{m}^{c}$ is the center of squirmer *m* and $u_{m}^{s}$ is the squirming velocity of squirmer *m*, defined by Equation (1).

It is supposed that squirmer *m* is subjected to known external forces **F***_m_* and torques **T***_m_*. The equilibrium conditions for squirmer *m* are:

(4) $$F_{m}=\int q\;dA_{m}\;,\;T_{m}=\int \left(r-r_{m}^{c}\right)\wedge q\;dA_{m}$$

**Figure 1:**
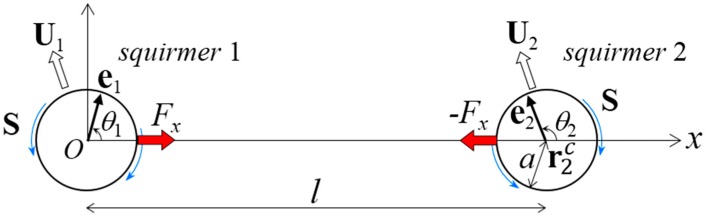
Problem settings for the far-field analysis. Two squirmers (1 and 2) with orientation vectors **e**_1_ and **e**_2_, respectively, are shown in the far field. The center of squirmer 1 was placed at the origin of coordinate. We assumed that **e**_1_ and **e**_2_ were in the *x–y* plane, where the *x*-axis was taken as the direction passing through the center of squirmer 2 at $r_{2}^{c}=\left(l,0,0\right)$ and *l* is the distance between the squirmer centers. This distance was assumed to be time-invariant with the application of *x*-direction force *F_x_*. No external torque was exerted; thus, the squirmer orientation was determined to satisfy the torque-free condition. The orientation angles *θ*_1_ and *θ*_1_ were defined from the *x*-axis. The radius of the squirmers was *a*, which satisfied *l* >> *a*. The squirmers swam with velocity **U***_i_*, where *i* was 1 or 2, and exerted a stresslet of **S**.

**Figure 2:**
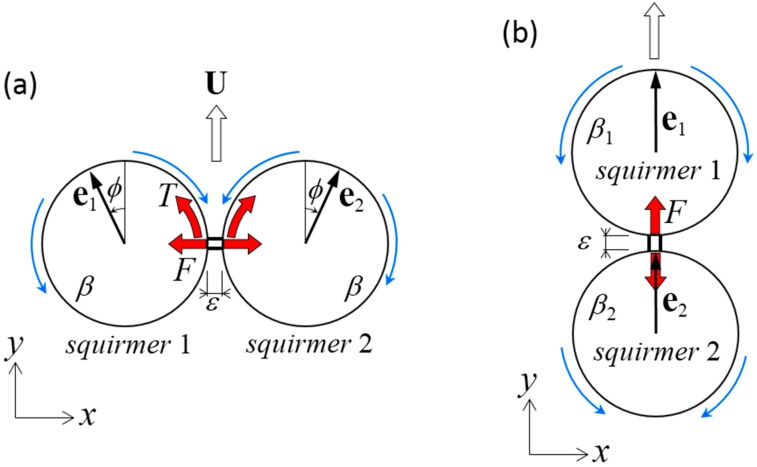
Problem settings for a dumbbell squirmer connected by a dragless rigid rod. The length of rod *ε* was set at 0.01*a*. (**a**) Two identical squirmers with swimming mode *β* were placed side-by-side at an angle of *ϕ* from the *y*-axis. Due to the symmetry of the problem, the dumbbell swam in the *y*-direction with velocity *U*. The rod imparted the *x*-direction force *F* and the *z*-direction torque *T* to the squirmers. (**b**) Two squirmers with swimming modes *β*_1_ and *β*_2_ were placed fore and aft. The squirmers were oriented in the *y*-direction and swam in the *y*-direction at velocity *U*. The rod exerted the *y*-direction force *F*.

We employed three different force–torque conditions in Section 3, Section 4 and Section 5:

(a) In Section 3, we assume that the distance between the centers of two far-field squirmers is invariant in time, i.e., *l* = const (Figure 1). In this case, **F***_m_* is generated so as to satisfy the constant distance condition. **T***_m_* is assumed to be zero, such that squirmer orientation satisfies the torque-free condition.
(b) In Section 4, we connect two near-field squirmers by a dragless rigid rod (Figure 2). In this case, both squirmers exhibit rigid body motion, i.e., $U_{2}=U_{1}+\Omega_{1}\wedge \left(r_{2}^{c}-r_{1}^{c}\right)$ and $\Omega_{2}=\Omega_{1}$. **F***_m_* and **T***_m_* are determined so as to achieve the rigid body motion.
(c) In Section 5, we connect two squirmer surfaces by a dragless linear spring (Figure 6). The spring is connected at $r_{m}^{s}$ on the surface of squirmer *m*, and generates force $F_{m}^{s}$ given by:
  (5) $$F_{1}^{s}=k\left(\left|r_{2}^{s}-r_{1}^{s}\right|-l_{0}\right)\frac{r_{2}^{s}-r_{1}^{s}}{\left|r_{2}^{s}-r_{1}^{s}\right|}\;,\;F_{2}^{s}=-F_{1}^{s}$$
  where *k* is the spring constant and *l*_0_ is the equilibrium length of the spring. The force generates a torque on squirmer *m* as:
  (6) $$T_{m}^{s}=\;\left(r_{m}^{s}-r_{m}^{c}\right)\wedge F_{m}^{s}$$

### 2.3. Numerical Methods

In Section 4 and Section 5, we simultaneously solve Equations (2) and (4) for the boundary condition (3). The governing equations are discretized using a boundary element method [31,40]. The details of these numerical methods are provided in our previous study [31]. Briefly, each spherical surface is discretized by 590 or 320 triangles, depending on the distance between the squirmers. The linear algebraic equations for the problem are generated by combining the governing equations and boundary condition. The integration is performed using 28-point Gaussian polynomials and the singularity is solved analytically. Time-marching is performed using a fourth-order Adams–Bashforth method.

## 3. Linear Stability Analysis of a Dumbbell Squirmer in the Far-Field

In this section, we investigate the stability of a dumbbell squirmer in the far field, because it can be clarified mathematically using linear stability analysis. We let squirmer 1 be at the coordinate origin (Figure 1), such that the *x*-axis passed through the center of squirmer 2 at (*l*, 0, 0). *l* was the center-to-center distance between the squirmers, and *a* was the squirmer radius; the far-field assumption requires that l>>a. The unit orientation vectors of squirmers 1 and 2 were **e**_1_ and **e**_2_, respectively. For simplicity, we assumed that both **e**_1_ and **e**_2_ existed in the same *x–y* plane. The angle of orientation vector **e***_m_* from the *x*-axis was *θ_m_*, where *m* is 1 or 2. To force dumbbell motion, the distance between the centers of the squirmers was set as invariant in time, i.e., *l* = const, by imposing *x*-direction force *F_x_* to squirmer 1 and −*F_x_* to squirmer 2. We imposed no external torque on the squirmers; therefore, squirmer orientation was determined so as to satisfy the torque-free condition. The torque condition can induce instability in the swimming of the dumbbell squirmer; we further investigated this phenomenon. Squirmer *m* swims with a translational velocity of **U***_m_* by generating surface squirming velocity. The squirming velocities were assumed to be the same for both squirmers, inducing the stresslet **S**, given by [31]:

(7) $$S=\frac{4}{3}\pi \mu a^{2}\left(3ee-I\right)B_{2}$$

where **I** is the identity matrix.

When force **F** and stresslet **S** are exerted on squirmer 1, rotational velocity *ω* far from the squirmer can be approximated as:

(8) $$\omega_{i}(r)=\frac{1}{16\pi \mu }\varepsilon_{ijk}\nabla_{j}\left(J_{kl}F_{l}+K_{klm}S_{lm}\right)$$

where **ε** is the alternating unit tensor. The kernel function **K** is given by [41]:

(9) $$K_{ijk}=\frac{1}{2}\left(\nabla_{k}J_{ij}+\nabla_{j}J_{ik}\right)$$

Equation (8) indicates that the *x*-direction force *F_x_* acting on squirmer 1 generates no rotational velocity at the center of squirmer 2, $r_{2}^{c}$. In contrast, the stresslet of squirmer 1 generates the following rotational velocity at $r_{2}^{c}$:

(10) $$\omega_{z}(r_{2}^{c})=\frac{1}{8\pi \mu }\frac{9}{2l^{3}}S_{0}\sin(2\theta_{1})$$

where $S_{0}=4\pi \mu a^{2}B_{2}/3$.

The translational velocity of squirmer 2 relative to squirmer 1 is **U**_2_ − **U**_1_, where the *x*-component is zero by definition. The *y*-component is non-zero, and can be approximated as $U_{0}\sin\left(\theta_{2}-\theta_{1}\right)$ in the leading order. The *y*-component velocities induce a rotational velocity of $U_{0}\left(\sin\theta_{1}-\sin\theta_{2}\right)/l$ to both squirmers relative to the center-to-center vector. Thus, the orientation change of the squirmers can be expressed as:

(11) $$\frac{d\theta_{1}}{dt}=\frac{U_{0}}{l}\left(\sin\theta_{1}-\sin\theta_{2}\right)+\frac{1}{8\pi \mu }\frac{9}{2l^{3}}S_{0}\sin(2\theta_{2})$$

(12) $$\frac{d\theta_{2}}{dt}=\frac{U_{0}}{l}\left(\sin\theta_{1}-\sin\theta_{2}\right)+\frac{1}{8\pi \mu }\frac{9}{2l^{3}}S_{0}\sin(2\theta_{1})$$

When the dumbbell squirmer swims in the steady state, the orientations are time-invariant. In this case, Equations (11) and (12) are solved as follows:

(13) $$\left(\;\theta_{1}^{*},\;\theta_{2}^{*}\right)=\left(\;0,\;0\right)\;,\;\left(\;0,\;\pi \right)\;,\;\left(\;\pi /2,\;\pi /2\right)\;,\;\left(\;\pi ,\;0\right)\;,\;\left(\;\pi ,\;\pi \right)$$

We now introduce small disturbances to the orientation angles, given by $\theta_{1}=\theta_{1}^{*}+\Delta \theta_{1}$ and $\theta_{2}=\theta_{2}^{*}+\Delta \theta_{2}$. By assuming that angle disturbances are sufficiently small, Equations (11) and (12) can be rewritten in the leading order as:

(14) $$\left(\begin{array}{c} \frac{d\Delta \theta_{1}}{dt}\\ \frac{d\Delta \theta_{2}}{dt} \end{array}\right)=\left(\begin{array}{cc} \frac{U_{0}}{l}\cos\theta_{1}^{*} & -\frac{U_{0}}{l}\cos\theta_{2}^{*}+\frac{9S_{0}}{8\pi \mu l^{3}}\cos(2\theta_{2}^{*})\\ \frac{U_{0}}{l}\cos\theta_{1}^{*}+\frac{9S_{0}}{8\pi \mu l^{3}}\cos(2\theta_{1}^{*}) & -\frac{U_{0}}{l}\cos\theta_{2}^{*} \end{array}\right)\left(\begin{array}{c} \Delta \theta_{1}\\ \Delta \theta_{2} \end{array}\right)$$

The eigenvalues of the matrix are:

(15) $$\lambda_{\pm }=\frac{U_{0}}{2l}\left(\cos\theta_{1}^{*}-\cos\theta_{2}^{*}\right)\pm \frac{1}{2}\sqrt{{\left\{\frac{U_{0}}{l}\left(\cos\theta_{1}^{*}+\cos\theta_{2}^{*}\right)\right\}}^{2}+4BC}$$

with

$$\begin{array}{cc} BC= & -{\left(\frac{U_{0}}{l}\right)}^{2}\cos\theta_{1}^{*}\cos\theta_{2}^{*}-\frac{U_{0}}{l}\cos\theta_{2}^{*}\frac{9S_{0}}{8\pi \mu l^{3}}\cos(2\theta_{1}^{*})\\ & +\frac{U_{0}}{l}\cos\theta_{1}^{*}\frac{9S_{0}}{8\pi \mu l^{3}}\cos(2\theta_{2}^{*})+{\left(\frac{9S_{0}}{8\pi \mu l^{3}}\right)}^{2}\cos(2\theta_{1}^{*})\cos(2\theta_{2}^{*}) \end{array}$$

Under the conditions of Equation (13), both eigenvalues become negative only when $\left(\;\theta_{1}^{*},\;\theta_{2}^{*}\right)=\left(\;\pi ,\;0\right)$ and *S*_0_ > 0. Thus, dumbbell puller squirmers can achieve stable swimming when they are oriented in opposite directions. In this case, however, the swimming velocity of the dumbbell squirmer is zero. These results indicate that the dumbbell squirmer cannot achieve stable forward swimming under the present problem settings, i.e., two squirmers in the far field with no external torque applied.

## 4. Swimming of a Dumbbell Squirmer Connected by a Short Rigid Rod

We next investigated the swimming of a dumbbell squirmer in the near field. When two squirmers are in the near field, the leading order contribution of the force and torque exerted on the squirmers can be derived mathematically using a lubrication theory [31]. However, next-order terms must be determined by matching the inner and outer solutions. Thus, a numerical approach may be necessary. To overcome this difficulty, we employed a boundary element method.

Before discussing the stability of the dumbbell squirmer in the near field, basic tendencies of dumbbell squirmer swimming must be clarified. We assumed that the squirmers were connected by a dragless short rigid rod (Figure 2) of length *ε* = 0.01*a*, although the value does not qualitatively change the results presented in this section. The dumbbell squirmer exhibits rigid body motion, i.e., $U_{2}=U_{1}+\Omega_{1}\wedge \left(r_{2}^{c}-r_{1}^{c}\right)$ and $\Omega_{2}=\Omega_{1}$, where **U***_m_* and **Ω***_m_* are the translational and rotational velocities, respectively, of squirmer *m*. To achieve rigid body motion, the connecting rod imparts forces **F***_m_* and torques **T***_m_* to the squirmers.

### 4.1. Two Identical Squirmers Connected Side-by-Side

We first investigated the swimming of two identical squirmers connected in parallel (Figure 2a). The dragless short rigid rod was connected at the minimum distance between two spherical surfaces. The squirmers had swimming mode *β* and angle *ϕ* from the *y*-axis. Due to the symmetry of the problem in the *y–z* plane, the dumbbell swam in the *y*-direction with velocity *U*. The rod imparted the *x*-direction force *F* and the *z*-direction torque *T* to the squirmers.

**Figure 3:**
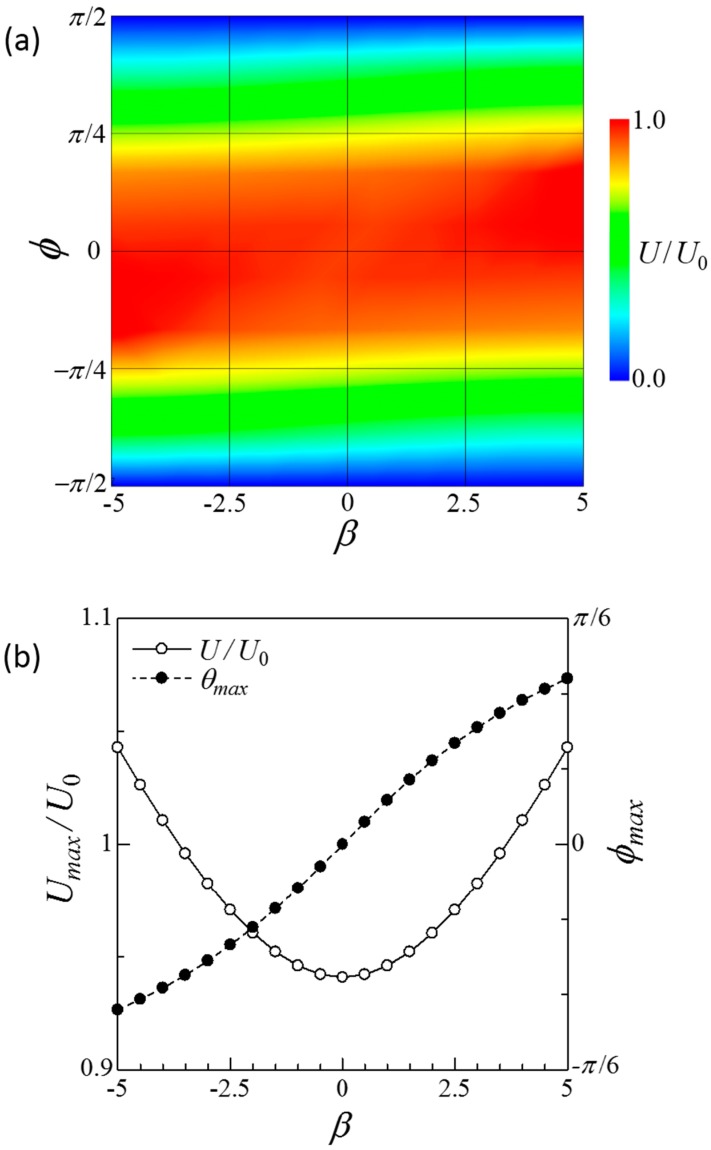
Swimming velocity of a side-by-side dumbbell squirmer connected by a short rigid rod. (**a**) Contour plot of the velocity in *β–ϕ* space. (**b**) Maximum velocity *U_max_* and the angle with maximum velocity *ϕ_max_* at each *β* value.

Figure 3 shows the swimming velocity of the side-by-side dumbbell squirmers. The velocity was nearly 1 when *ϕ* = 0, i.e., parallel orientation, whereas it was zero when *ϕ* = ±π. Figure 3b shows that maximum swimming velocity slightly increased as |*β*| increased. The angle with maximum velocity *ϕ_max_* increased as *β* increased. These results indicate that swimming velocity was not significantly increased in the case of the side-by-side dumbbell squirmers.

**Figure 4:**
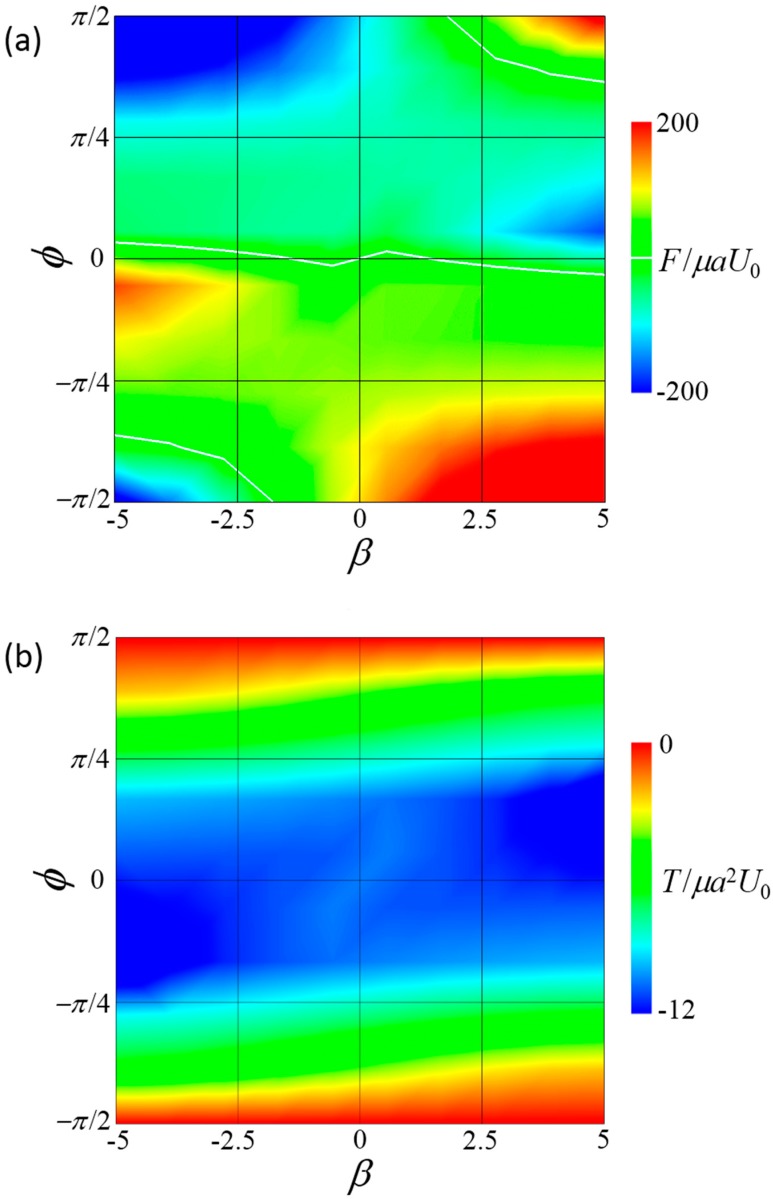
Force *F* and torque *T* induced by the connecting rod of the side-by-side dumbbell squirmers. (**a**) Contour plot of the force in *β–ϕ* space, where negative *F* indicates that the squirmers tended to repel each other without the rod. White lines indicate *F* = 0. (**b**) Contour plot of torque in *β–ϕ* space, where negative *T* indicates that the squirmers tended to turn away from each other without the rod.

Figure 4 shows the force *F* and torque *T* induced by the connecting rod. When *F* was positive, two squirmers tended to attract each other and the rod generated force, causing the squirmers to repel each other. When *T* was positive, two squirmers tended to face each other and the rod generated torque to face them in the opposite direction. The force was strongly affected by both *β* and *ϕ*, and changed its sign depending on these parameters. In contrast, torque was always negative in the range −5≤β≤5. When torque was negative, the squirmers tended to orient away from each other and the rod generated torque to turn them face to face. These results show that steady forward swimming cannot be achieved in a side-by-side dumbbell squirmer without the application of external torque to counterbalance the hydrodynamic torque.

### 4.2. Squirmers with Different Modes Connected Fore and Aft

We next investigated the swimming of two squirmers with different squirming modes, *β*_1_ and *β*_2_, connected fore and aft (Figure 2b). A dragless short rigid rod was again connected at the minimum distance between the spherical surfaces. The two squirmer centers were placed along a line parallel to the *y*-axis, and both squirmers were oriented in the *y*-direction. Due to symmetry of the problem along the *y*-axis, the dumbbell swam in the *y*-direction with velocity *U*. The rod exerted *y*-direction force *F*, and no torque was generated on the squirmers.

**Figure 5:**
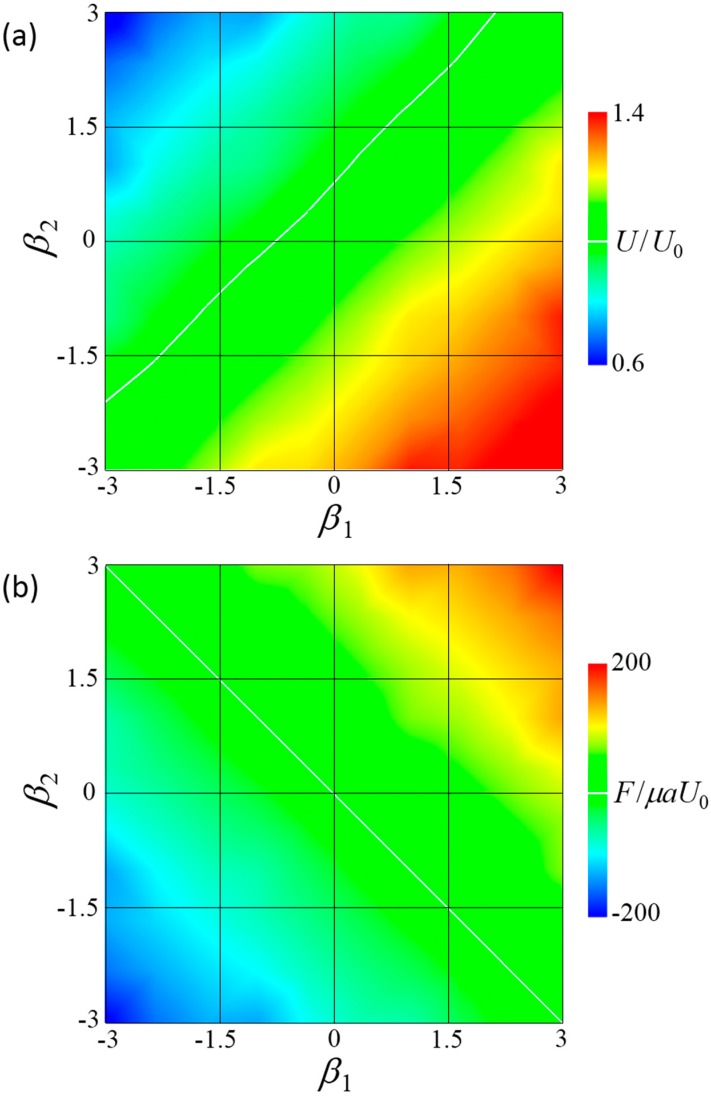
Swimming behavior of a fore and aft dumbbell squirmer connected by a short rigid rod. (**a**) Contour plot of velocity at various swimming modes. White line indicates *U/U*_0_ = 1. (**b**) Contour plot of force at various swimming modes, where negative *F* indicates that the squirmers tended to repel each other without the rod. White line indicates *F* = 0.

Figure 5a shows the swimming velocity of the fore-and-aft dumbbell squirmer. The velocity differed considerably depending on the squirming mode, with a significantly greater velocity observed when the fore squirmer was a puller and the aft squirmer a pusher, i.e., *β*_1_ > 0 and *β*_2_ < 0. When *β*_1_ = 3 and *β*_2_ = −3, velocity increased to become about 1.4 times larger than that of a solitary squirmer. When *β*_1_ = −3 and *β*_2_ = 3, velocity decreased to become about 0.6 times smaller than that of a solitary squirmer.

Figure 5b shows force *F* induced by the connecting rod. When *F* was positive, the squirmers tended to attract each other and the rod generated a repelling force between the squirmers. The sign of the force changed along *β*_1_ + *β*_2_ = 0. When *β*_1_ + *β*_2_ > 0, *F* was positive and the squirmers tended to attract each other. When *β*_1_ + *β*_2_ < 0, *F* was negative and the squirmers tended to repel each other.

In designing a real dumbbell swimmer, the two micro-swimmers can be connected by a flexible string instead of a connecting rigid rod. Such a string cannot resist compression force, because it can be bent easily. However, stretching force can be effective because the string can generate a contraction force when fully stretched, by setting *β*_1_ + *β*_2_ < 0.

## 5. Stability of a Dumbbell Squirmer Connected by a Spring

In considering real applications of assembled micro-swimmers, it is preferable to connect or detach swimmers in a reversible manner, which can be achieved using the reversible adhesion of proteins or polymer chains. Since the mechanical behavior of these macromolecules is similar to that of a spring, the swimmer can be modeled as a dumbbell micro-swimmer connected by a spring. Moreover, the rigid connecting rod used in Section 4 may be too ideal. The rod should have some elasticity, and may be bent at the base, i.e., on the squirmer surface. A dumbbell micro-swimmer connected by a spring may be a more realistic model; therefore, we investigated the stability of a dumbbell squirmer connected by a linear spring.

### 5.1. Two Identical Squirmers Are Connected Side-by-Side

**Figure 6:**
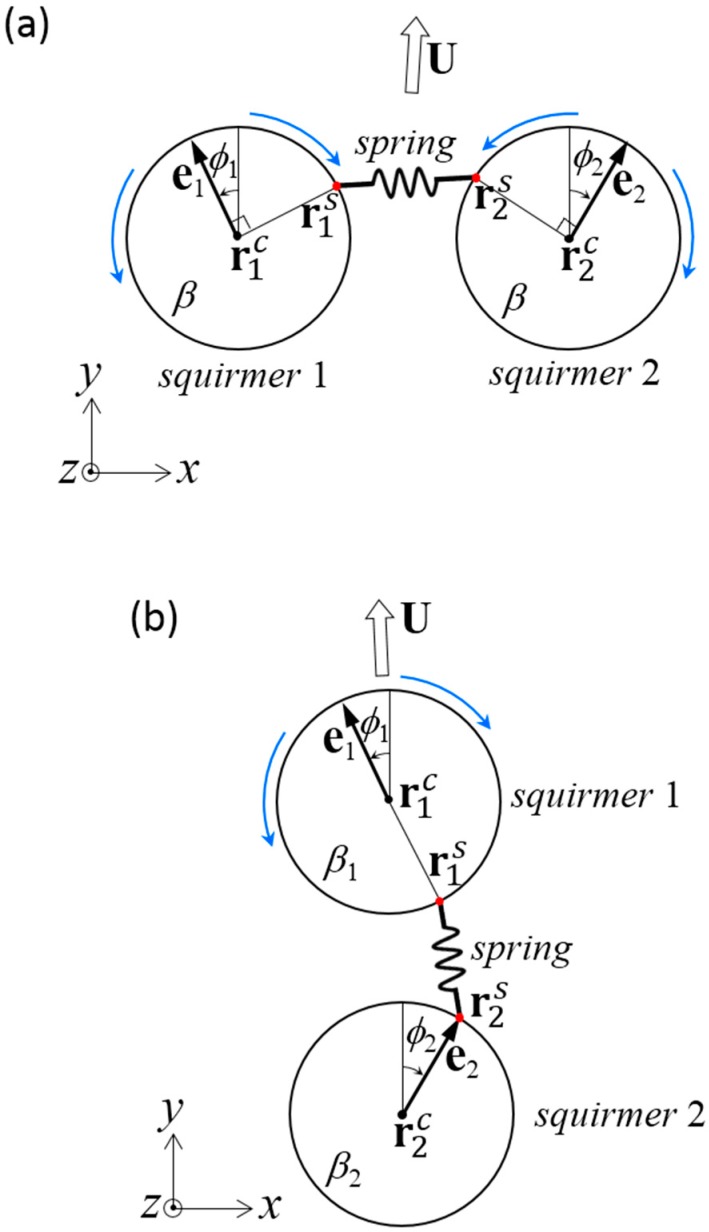
Problem settings for a dumbbell squirmer connected by a spring. The spring was connected between $r_{1}^{s}$ and $r_{2}^{s}$ on the surface of squirmer 1 and 2, respectively. Squirmer *i* had orientation vector **e***_i_*, squirming mode *β_i_*, angle from the *y*-axis *ϕ_i_*, and the center position $r_{i}^{c}$. (**a**) Two identical squirmers with swimming mode *β* were connected side-by-side. (**b**) Two squirmers connected fore and aft.

We first investigated the swimming behavior of two identical squirmers connected in parallel by a dragless linear spring (Figure 6a). The spring was connected at $r_{m}^{s}$ on the surface of squirmer *m*, where $r_{m}^{s}$ was on the equator. We assume that the spring can change angle at $r_{m}^{s}$ without any friction. The spring generated force given by Equation (5), where the equilibrium length was set as *l*_0_ = *a*. The spring constant *k* was set as *k*/*μU*_0_ = 100, to ensure that the spring was sufficiently strong and its length did not change considerably. The spring force generated torque on the squirmer, as shown in Equation (6). The squirmers had the same swimming mode *β*.

To introduce small disturbances and asymmetry in the squirmer configuration, the centers of squirmers 1 and 2 were initially placed at $r_{1}^{c}=\left(\;0,\;0,\;0\right)$ and $r_{2}^{c}=\left(3a,0,0.1a\right)$. The squirmers were oriented in the *x–y* plane, at an angle from the *y*-axis described by $e_{1}=\left(-\sin\varphi_{1},\;\cos\varphi_{1},\;0\right)$ and $e_{2}=\left(\;\sin\varphi_{2},\;\cos\varphi_{2},\;0\right)$. We set *ϕ*_1_ = 1° and *ϕ*_2_ = 2°. The dumbbell squirmer swam with velocity **U**. We varied swimming mode *β*, and examined the stability of side-by-side swimming. We observed that stability was not affected by the magnitude of the initial disturbance, provided that the disturbance was not too large.

**Figure 7:**
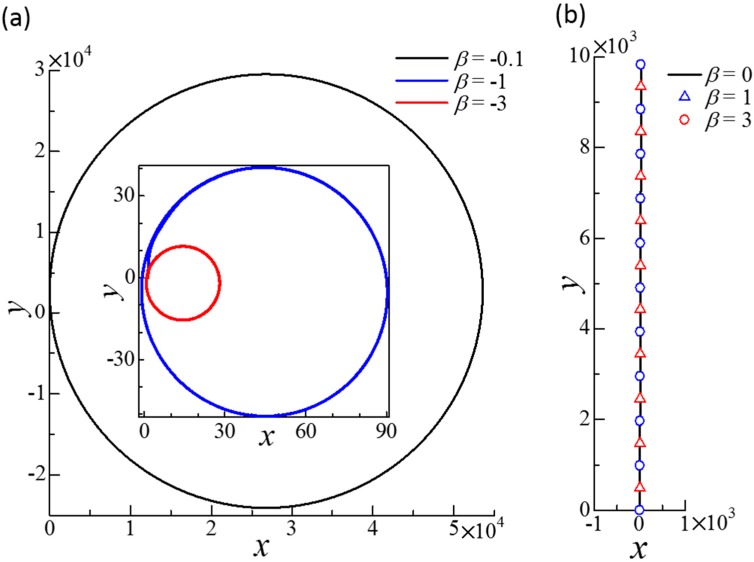
Effect of *β* on the trajectories of the squirmer centers of dumbbell squirmers connected side-by-side. To generate a small disturbance, two squirmers were initially placed at $r_{1}^{c}$ = (0, 0, 0) and $r_{2}^{c}$ = (3*a*, 0, 0.1*a*) with *ϕ*_1_ = 1° and *ϕ*_2_ = 2°. The equilibrium length of the spring was set at *a*. (**a**) Circular trajectories obtained in negative *β* cases. Inset shows the cases *β* = −1 and −3. (**b**) Straight trajectories obtained for *β* = 0, 1, and 3.

Figure 7 shows the trajectories of dumbbell squirmers connected in parallel with a spring. Trajectories were circular when *β* was negative. Squirmers with negative *β* became stable with inclined squirmer orientation, such that the dumbbell generated rotational velocity as well as translational velocity. When β≥0, the paths of the dumbbell squirmer were straight. In this case, the squirmers eventually became almost parallel to each other, and their direction almost coincided with the swimming direction. These results indicate that the side-by-side dumbbell was stable when the squirmers were pullers. The swimming speed of the dumbbell squirmer was almost *U*_0_, such that side-by-side swimming did not greatly affect swimming speed (Figure 3).

**Figure 8:**
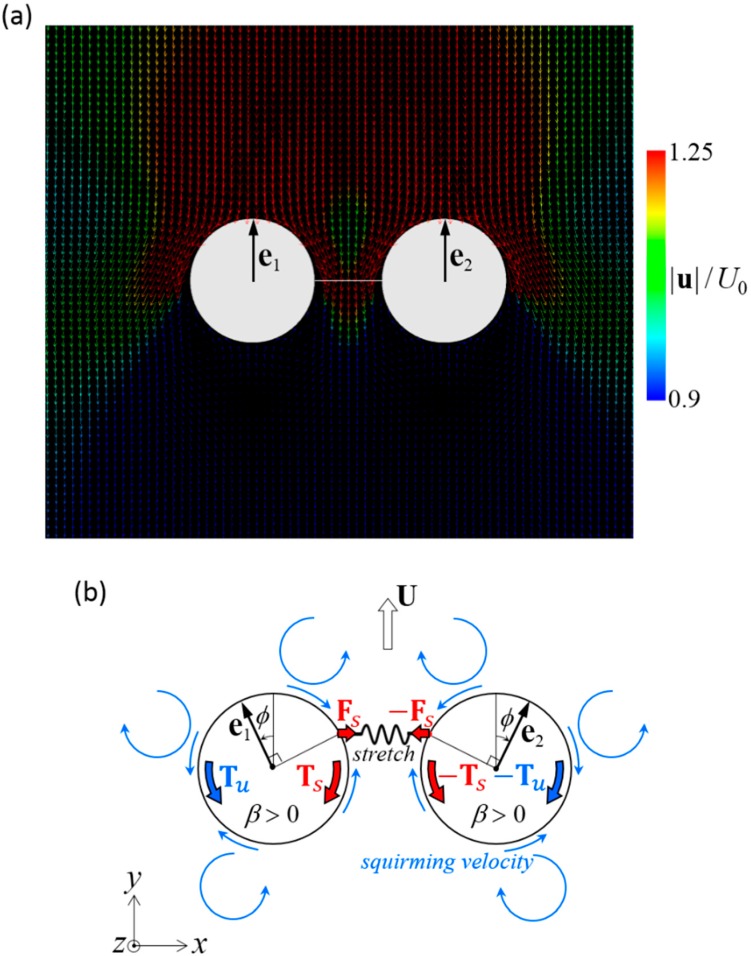
Mechanism of stable swimming of dumbbell squirmers connected side-by-side. (**a**) Velocity field around a stable dumbbell squirmer with *β* = 3. (**b**) Schematics of the torque balance. Two squirmers tended to move away from each other, and the spring was stretched. The spring force **F***_s_* induced spring torque **T***_s_* to the squirmer (red arrows). The squirming velocity (blue arrows), generated hydrodynamic torque **T***_u_*. The configuration became stable when the two torques canceled each other out.

The mechanism of stability can be explained by the torque balance of the squirmers. Figure 8a shows the velocity field around a stable side-by-side dumbbell squirmer with *β* = 3. A large velocity was generated in front of the squirmer and a small velocity was generated behind the squirmer, where recirculation regions were observed. The orientation vectors appear almost parallel to each other, but slightly apart, due to the hydrodynamic torque exerted in the side-by-side orientation (Figure 4b). The velocity field generated by pullers is shown schematically in Figure 8b. Since the squirmers were slightly apart from each other, the connecting spring was stretched, imparting force **F***_s_* to squirmer 1 and resulting in spring torque **T***_s_* (red arrow). This torque counterbalanced hydrodynamic torque **T***_u_*, which was generated by the squirming velocity of squirmer 2. Since the torques canceled each other out, the squirmer was able to maintain steady orientation while swimming.

### 5.2. Squirmers with Different Modes Connected Fore and Aft

We next investigated swimming of two squirmers with different squirming modes *β*_1_ and *β*_2_, connected fore and aft by a dragless linear spring (Figure 6b). The spring connected the bottom of squirmer 1 ($r_{1}^{s}=r_{1}^{c}+ae_{1}$) and the top of squirmer 2 ($r_{2}^{s}=r_{2}^{c}+ae_{2}$). The spring generated force and torque as given by Equations (5) and (6), at an equilibrium length of *l*_0_ = *a* and spring constant of *k*/*μU*_0_ = 100.

To introduce small disturbances and asymmetry in the squirmer configuration, the centers of squirmers 1 and 2 were initially placed at $r_{1}^{c}=\left(\;0,\;0,\;0\right)$ and $r_{2}^{c}=(0,-3a,0.1a)$. The squirmer vectors were oriented in the *x*–*y* plane; the angles from the *y*-axis were $e_{1}=\left(\;-\sin\varphi_{1},\;\cos\varphi_{1},\;0\right)$ and $e_{2}=\left(\;\sin\varphi_{2},\;\cos\varphi_{2},\;0\right)$. We set *ϕ*_1_ = 1° and *ϕ*_2_ = 2°. The dumbbell squirmer swam at velocity **U**. To reduce the number of parameters, we assumed that *β*_1_ = 0 and varied swimming mode *β*_2_ to examine the stability of fore and aft swimming. We again observed that stability was not affected by the magnitude of the initial disturbance, provided that the disturbance was not too large.

**Figure 9:**
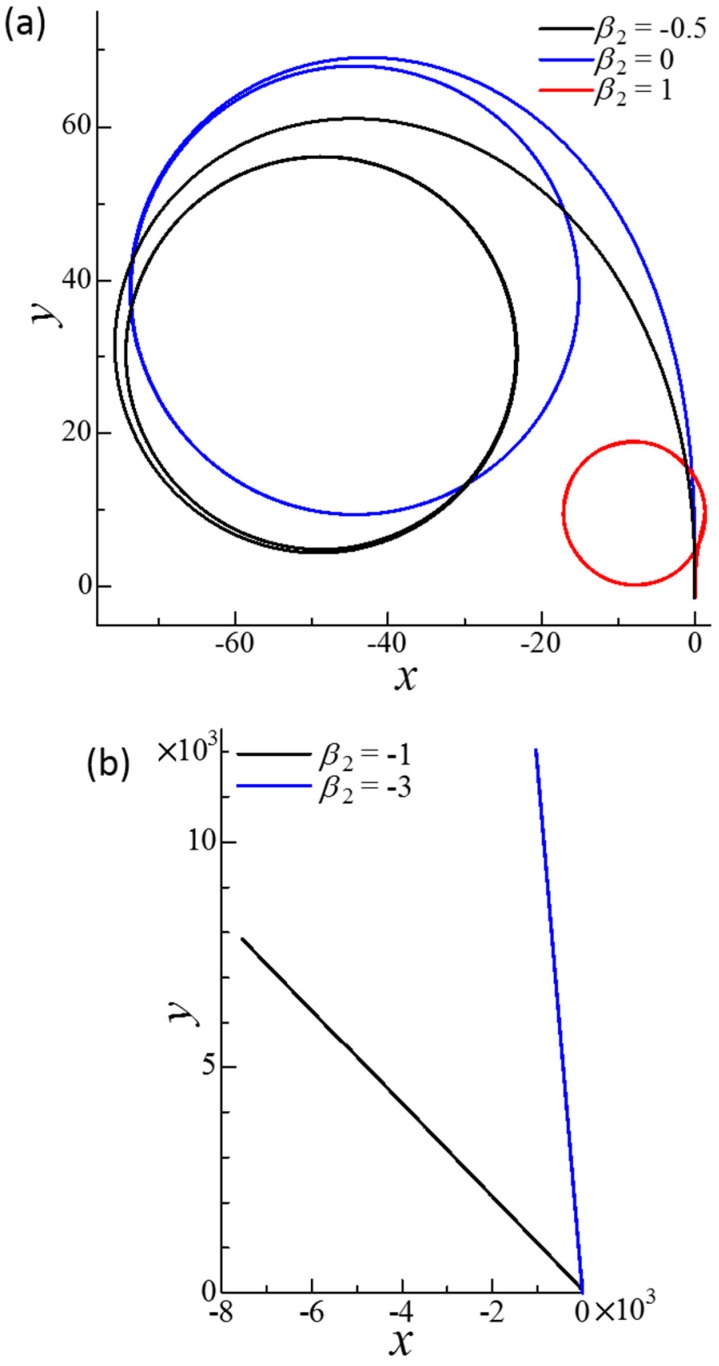
Effect of *β*_2_ on the trajectories of the squirmer centers of the dumbbell squirmer connected fore and aft. The squirming mode of squirmer 1 was *β*_1_ = 0. The squirmers were initially placed at $r_{1}^{c}$ = (0, 0, 0) and $r_{2}^{c}$ = (0, −3*a*, 0.1*a*) with *ϕ*_1_ = 1° and *ϕ*_2_ = 2° to generate a small disturbance. The equilibrium length of the spring was *a*. (**a**) Circular trajectories obtained with *β*_2_ = −0.5, 0, and 1. (**b**) Straight trajectories obtained with *β*_2_ = −1 and −3.

Figure 9 shows the trajectories of the dumbbell squirmers connected fore and aft by the spring. Trajectories with $\beta_{2}\geq -0.5$ eventually became circular, although convergence was slow. In these cases, the squirmers became stable at inclined orientations, such that the dumbbell generated rotational as well as translational velocity. When $\beta_{2}\leq -1$, the dumbbell squirmer paths were straight. The swimming directions did not coincide with the *y*-direction, because the initial disturbance greatly changed the swimming direction within the first tens of time unit. These results demonstrate that the fore and aft dumbbell was stable when the aft squirmer was a strong pusher. The swimming speed of the dumbbell squirmer at *β*_2_ = −3 was about 1.2*U*_0_, indicating that the fore and aft squirmers were able to increase swimming speed considerably (Figure 5a).

**Figure 10:**
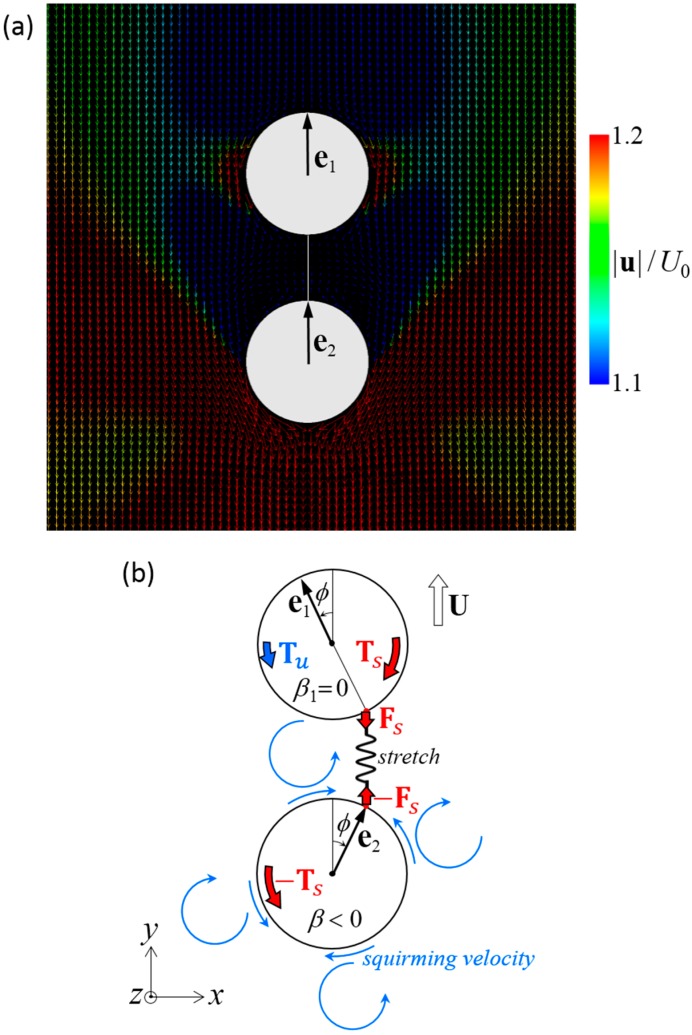
Mechanism of the stable swimming in a dumbbell squirmer connected fore and aft. (**a**) Velocity field around a stable dumbbell squirmer with *β*_1_ = 0 and *β*_2_ = −3. (**b**) Schematics of the torque balance. Two squirmers tended to repel each other, and the spring was stretched. Spring force **F***_s_* induces spring torque **T***_s_* to the squirmer (red arrows). The spring torque stabilized the aligned configuration when it overwhelmed hydrodynamic torque **T***_u_*, which was caused by the squirming velocity.

The mechanism of stability can again be explained by the torque balance of the squirmers. Figure 10a shows the velocity field around a stable fore and aft dumbbell squirmer with *β*_1_ = 0 and *β*_2_ = −3. A large velocity was generated behind the aft squirmer, whereas a small velocity was generated in front of the fore squirmer and between the squirmers. In this case, the orientation vectors were aligned. The velocity field around the dumbbell squirmers is shown schematically in Figure 10b. Since the aft squirmer was a strong pusher, the squirmers tended to repel each other (Figure 5b). The repulsion flow stretched the connecting spring, imparting force **F***_s_* to the squirmers. The spring force resulted in spring torques **T***_s_* (red arrows), which overwhelmed the hydrodynamic torques **T***_u_* generated by the squirming velocities; thus, the two squirmers eventually aligned.

## 6. Conclusions

We investigated the swimming behavior and stability of a dumbbell squirmer. We first investigated stability in the far field using linear stability analysis. The results indicated that stable forward swimming could not be achieved when the two squirmers were in the far field without the application of external torque. We then investigated the swimming speed of a dumbbell squirmer connected by a short rigid rod using a boundary element method. Two types of dumbbell squirmers were examined: (a) side by side and (b) fore and aft. A significant increase in the swimming velocity was observed when the fore squirmer was a puller and the aft squirmer was a pusher. The force and torque exerted by the dumbbell squirmer were also clarified. Finally, we investigated the swimming stability of a dumbbell squirmer connected by a spring. We demonstrated that stable side-by-side swimming could be achieved by pullers. Fore and aft swimming were also stable when the aft squirmer was a strong pusher, such that the swimming speed was significantly increased. The findings of this study form a fundamental basis for micro-swimmer mechanics and will be useful in creating future designs of assembled micro-swimmers.
